# The Efficacy of Sitz Baths as Compared to Lateral Internal Sphincterotomy in Patients with Anal Fissures: A Systematic Review

**DOI:** 10.7759/cureus.30847

**Published:** 2022-10-29

**Authors:** Ali R Alnasser, Aqsa Akram, Saikat Kar, Fatema Osman, Ghadi D Mashat, Hadrian Hoang-Vu Tran, Neway A Urgessa, Prabhitha Geethakumari, Prathima Kampa, Rakesh Parchuri, Renu Bhandari, Ann Kashmer Yu

**Affiliations:** 1 General Surgery, California Institute of Behavioral Neurosciences & Psychology, Fairfield, USA; 2 Internal Medicine, Dallah Hospital, Riyadh, SAU; 3 Internal Medicine, California Institute of Behavioral Neurosciences & Psychology, Fairfield, USA; 4 Pediatrics, California Institute of Behavioral Neurosciences & Psychology, Fairfield, USA; 5 Pathology, California Institute of Behavioral Neurosciences & Psychology, Fairfield, USA; 6 Internal Medicine, California institute of Behavioral Neurosciences & Psycholgy, Fairfield, USA; 7 Internal Medicine, Manipal College of Medical Sciences, Kaski, NPL; 8 Internal Medicine/Family Medicine, California Institute of Behavioral Neurosciences & Psychology, Fairfield, USA

**Keywords:** piles, lateral internal sphincterotomy, surgical management, conservative management, anal fissures, sitz bath

## Abstract

An anal fissure is a common condition that affects patients of all ages. Its clinical presentation is a sharp pain on defecation with or without blood. It is treated by conservative or surgical means. This study aims to assess the efficacy of a sitz bath as compared to lateral internal sphincterotomy in the treatment of anal fissures. The search strategy used keywords related to the topic of study. Three databases were used: PubMed, Google Scholar, and Science Direct. A total of 551 articles were screened. A quality assessment check was done on the articles leaving 11 articles. Four aspects of sitz bath outcomes were evaluated in the articles. In terms of analgesia, articles showed conflicting evidence. However, the overall evidence supports the use of sitz baths for their analgesic properties. In terms of healing, most articles had similar recovery rates of around 80%. Much of the research supported the use of sitz baths as the primary treatment to heal acute fissures. When compared to lateral internal sphincterotomy, the recovery rates of lateral internal sphincterotomy are superior to those of conservative treatment, including sitz baths. However, studies showed incontinence as a side effect of lateral internal sphincterotomy, and no studies reported side effects from the sitz baths. To conclude, the results of the articles support the use of sitz baths to treat anal fissures. Sitz baths have been found to have analgesic properties, as well as a good healing time. But, compared to lateral internal sphincterotomy, there is a significant difference in the healing rate at the end stage of treatment, lateral internal sphincterotomy is found to be superior. With regards to the side effects, none have been reported from using a sitz bath.

## Introduction and background

Anal fissures are a break in the skin located below the dentate line of the anal canal. It typically involves the lower portion of the internal sphincter. It presents with a sharp pain when passing stool, associated with streaks of blood in the stool. Fissures occur as a result of trauma to the anal canal. It usually occurs due to overstretching of the underlying skin. Pathologic manifestations may include a sentinel pile or a hypertrophied papilla. Typically, fissures are categorized into acute fissures that heal within six weeks and chronic fissures that last more than six weeks [1].

The management of anal fissures is extensive, using a primarily conservative approach, then medically, leading up to surgical intervention. Up to 87% of anal fissures resolve with conservative treatments, including sitz baths, stool softeners, and ointments [2]. Adequate fluid intake and high-fiber diets are also used to treat fissures. Providing heat to the fissure area has also been shown to relieve the stress of the hypertonic sphincter.

Conservative treatment is considered a low-risk treatment with fewer recurrences and is cost-effective. Medical therapies in use include glyceryl trinitrate (GTN) and topical calcium channel blockers that work by reducing cytosolic calcium, causing smooth muscle relaxation. Diltiazem and nifedipine are used as calcium channel-blocking agents and have been shown to heal 65-95% of anal fissures. These agents can also be combined with other conservative treatment modalities. The botulinum toxin is another medical intervention used to treat fissures. This is considered chemical sphincterotomy, leading to resolution in up to 80% of cases with a single injection. Healing takes longer than surgical methods, but the risks of incontinence are almost zero.

Surgical treatments are common when dealing with chronic fissures. Lateral internal sphincterotomy (LIS) is the gold standard of treatment, leading to a resolution in 95% of cases, with faster healing and the least number of risks as compared to a fissurectomy. An alternative to lateral internal sphincterotomy is anal advancement flap surgery, in which a flap of skin with good vascularization is advanced into the sphincter after the fissure is removed. This method lacks randomized studies. Anal dilatation is a less preferred modality of treating anal fissures as it is associated with higher incontinence and recurrence rates [3].

This systematic review will focus on the importance of conservative management and the effectiveness of sitz baths in a population suffering from anal fissures as compared to the gold standard of lateral internal sphincterotomy surgical management. This research aims to find pragmatic solutions to a common condition, taking into consideration the complications, risks, and effectiveness of the treatment. Anal fissures affect a large and diverse population of all ages. A conservative approach should be explored and should be considered for all cases before prioritizing the gold-standard surgical treatment [4]. Currently, only a few studies have looked into the effectiveness of sitz baths in treating anal fissures. This review aims to collect the available data and provide a rationale for the use of prescribing sitz baths. This review appreciates that sitz baths alone are not prescribed to treat anal fissures and explores the rationale and evidence for prescribing them with or without adjuncts.

## Review

Methods

This systematic review was conducted based on the Preferred Reporting Items for Systematic Reviews and Meta-Analyses (PRISMA) 2020 guidelines [5].

Eligibility Criteria

The inclusion criteria used in this systematic review included keywords related to three concepts in relation to the topic. Further criteria were added to narrow down the relevant articles. Articles published between 1983 and 2022; only human studies; only English and Arabic; free text; randomized control trials (RCT); cohorts; and systematic were included. Membership access literature, books, editorials, unpublished work, and gray literature were excluded.

Search Strategy

There were a limited number of publications regarding the topic. Hence, a search strategy was developed to include more articles that were relevant to the review. This is shown in Table 1.

**Table 1:** The databases and search strategy used in the systematic review

Databases	Keywords	Search strategy	Filters	Search results
PubMed	anal fissure, fissure-in-ano, rectal fissure, sitz bath, hip bath, therapeutic bath, hot tub, conservative, noninvasive, nonsurgical	#1 anal fissure OR Fissure-in-ano OR rectal fissure OR "Fissure in Ano"[Majr] #2 sitz bath OR hip bath OR therapeutic bath OR hot tub OR ( "Baths/therapeutic use"[Majr] OR "Baths/therapy"[Majr] ) #3 conservative or noninvasive or nonsurgical ( "Conservative Treatment/therapeutic use"[Majr] OR "Conservative Treatment/therapy" [Majr] ) #4 deleted #5 #1 and #2 #6 #1 and #3 #7 #5 or #6 #7 was finally used to search for the articles	Dates: 1992 - 2022 Languages: English and Arabic full-text	197
Science Direct	anal fissure, sitz bath	anal fissure and sitz bath	Dates: 1992 - 2022	104
Google Scholar	anal fissure, sitz bath	anal fissure and sitz bath	Dates: 1992 - 2022	2230 (only the first 297 articles were included)

Using keywords from the three concepts of the topic, the MeSH vocabulary system was used. The first search was conducted through PubMed. All articles from the search were exported to an excel document. Initially, the articles were filtered out based on the relevancy of the article titles, leaving only articles with keywords in the title. Articles with irrelevant titles were excluded. Then, the articles were further filtered out based on the abstract, leaving in any article featuring conservative treatments of anal fissures. Articles with irrelevant abstracts were excluded. Then, the articles were filtered out based on the relevancy of their content. Articles featuring sitz baths as part of the conservative treatment were included. Articles focusing on other anorectal disorders were excluded. Lastly, all editorials and gray literature articles were excluded. This was done to focus on stronger forms of research, including randomized control trials, cohorts, and systematic reviews.

Methodological Quality Assessment 

Various types of quality assessment tools were used for various types of research. The Cochrane Bias Assessment Tool (Rob2) was used for randomized control trials. Newcastle Ottawa Toll was used for non-randomized trials and observational studies. The Assessment of Multiple Systematic Reviews (AMSTAR) was used for systematic reviews. The Scale for the Quality Assessment of Narrative Review Articles (SANRA) was used for other studies [6-9]. All scores were converted to a percentage, and articles that scored less than 60% were excluded.

Results

Study Selection

**Figure 1:**
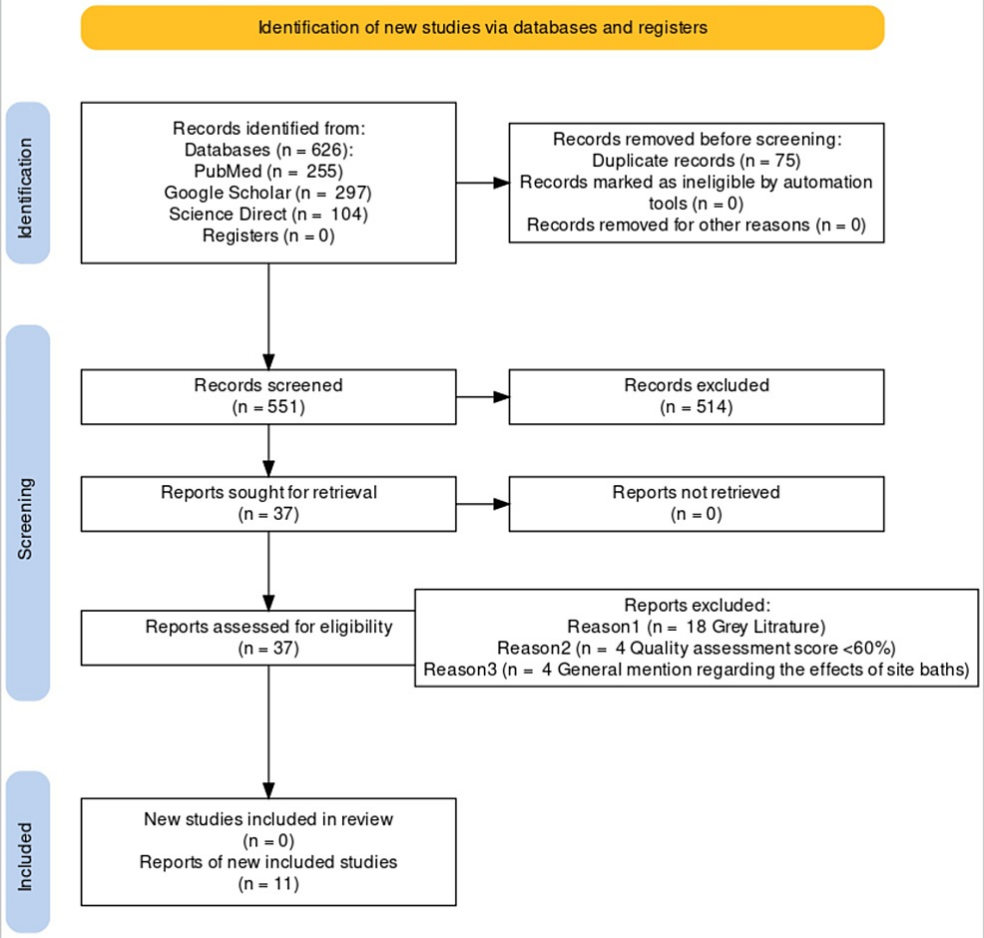
Preferred Reporting Items for Systematic Reviews and Meta-Analyses (PRISMA) flow diagram for the selection of articles.

As seen in Figure 1 [10], the PRISMA Flow Diagram shows the three databases used (PubMed, Google Scholar, and Science Direct). A total of 626 articles were extracted and listed in an excel document. The articles were sorted alphabetically, and all duplicates were removed. After removing 75 duplicates, 551 results were left for screening. Then, the articles were screened using the keywords mentioned above, leaving 37 relevant articles. From there, 18 articles were excluded due to their being grey literature. According to the type of study, the appropriate quality assessment tool was utilized. Each article was then given a quality assessment score. Four articles had a score of less than 60%. Four articles were removed for not detailing the effect of sitz baths in the treatment of anal fissures, leaving 11 articles to qualify. Both the main author and co-author were involved in the selection of each article.

Methodological Quality

Five randomized control trials were included in the review. They were all assessed using the Cochrane Assessment Tool. Participants in all trials were randomly assigned to a treatment group. Most studies were not blinded. However, the risk of bias was low in all selected randomized control trials. Five non-randomized control trials were included in this review. They were all assessed using the Newcastle Ottawa Tool. The trial and control groups were both comparable in all studies. One systematic review was included and assessed using the AMSTAR 2 guidelines. The systematic review was based on four randomized control trials. The articles were assessed using the Joanna Briggs Institute (JBI) Assessment Tool.

Data Synthesis

Since the articles were significantly heterogeneous, statistical pooling could not be done. Instead, the results are displayed in narrative form. Table 2 shows the key points extracted from each article.

**Table 2:** Key points extracted from each article for the systematic review. RCT: Randomised clinical trial, GTN: Glyceryl trinitrate, LIS: Lateral internal sphincterotomy, LSIAS: Lateral subcutaneous internal anal sphincterotomy

Author	Type of Study	Population	Country	Age	Outcome	Conclusion	
Gogna S et al., 2015 [11].	RCT	75	-	35.5-32.4	Patients were divided into three treatment groups, and their healing rates were recorded in three weeks. Glyceryl trinitrate (GTN): 84%, Sitz bath: 80%, Lignocaine: 40%.	A Sitz bath can be equally effective as GTN in the treatment of an acute anal fissure. Lignocaine has a lower efficacy in the management of acute anal fissures.	
Jensen SL et al., 1983 [12].	RCT	96	Denmark	-	Over three weeks of treatment, the healing rate was monitored in three groups: In the Lignocaine group, 20%, 40%, and 60% healed in the first, second, and third weeks, respectively. In the hydrocortisone group, 32.4%, 64.7%, and 82.4% healed in the first, second, and third weeks, respectively. Warm sitz bath plus bran group: 46.9%, 75%, and 87.5% healed in the first, second, and third weeks, respectively.	Patients with the first episode of acute anal fissure can be symptomatically managed by warm sitz baths and with a dietary intake of unprocessed bran.	
Maestre Y et al., 2010 [13].	RCT	24	-	-	There were no statistical differences in pain, but it remained constant in the cold sitz bath group and gradually decreased in the warm sitz bath group.	There was no statistical difference in cold vs. hot sitz baths.	
Rathore RK et al., 2019 [14].	Non-RCT	50	India	31-40	Patients were divided into two groups and used a visual analog scale to score the pain 0-5. 0 indicates no improvement, and 5 indicates excellent improvement. The mean scores are as follows. Sitz bath: 1.65, 2.05, 2.76 on the third, fifth, and seventh day, respectively. Sitz shower: 2.12, 3.43, and 4.65 on the third, fifth, and seventh day, respectively.	A warm sitz shower bath displayed a noticeable outcome in terms of pain relief, improvement in symptoms, and satisfaction, similar to patients receiving the warm sitz bath.	
Lang DS et al., 2010 [15].	Systematic review	280	Singapore	18-75	Four RCTs were used. Two treatment groups were made and the percentage of patients recovering from each study was recorded. With sitz bath: 85.1%, 95%, 95% in Gupta 2006, Gupta 2007, and Gupta 2008. Without sitz bath: 84%, 93%, 94% in Gupta 2006, Gupta 2007, and Gupta 2008.	This review does not yield convincing evidence in support of the use of sitz baths in patients with anorectal disorders for pain relief or to hasten wound healing. No complications were reported with the use of sitz baths. The study concludes that the use of sitz baths is dependent on patient satisfaction. The study suggests that water spray maintains hygiene.	
Motie MR et al., 2015 [16].	RCT	190	Iran	16-68	Patients were put into three treatment groups: Sitz bath + 3% nitroglycerin: 74% healing after four and eight weeks; Sitz bath + diltiazem: 25%, 83% healing after four and eight weeks; LIS: 70%, 94% healing after four and eight weeks, respectively.	Side effects were noted. Nitroglycerin use resulted in headaches; no side effects were reported with diltiazem use; LIS-3% showed gas and liquid incontinence that spontaneously resolved in four weeks.	
Kaushal M et al., 2022 [17].	RCT	90	India	25-53.1	Patients were divided into three treatment groups, and treatment recovery rates and complications were monitored. The surgical group had 95-100% achieved full recovery. Three had wound infections. Two had incontinence flatus. The Zinc group had a recovery rate of 23.3%, while the Lidocaine group had a recovery rate of 26.6%. Of all patients, 71/90 showed increased anal tone.	90 patients were randomly assigned to three groups: surgical, lidocaine, and zinc. Patients with zinc oxide pomade with sitz bath showed a 23.33% recovery rate. A 26.66% recovery rate was shown for Lidocaine pomade with a hot sitz bath. LIS showed a 99–100% recovery rate. There was no significant difference in recovery rates between non-operative treatments.	
Emile SH et al., 2017 [18].	Non-RCT	65	Egypt	16-73	The duration of symptoms impacts response. Acute vs. chronic fissures.	Chronic fissures often do not resolve with conservative treatment. Acute fissures have a good response. Healing was 100% if symptoms started more than one month ago. Then it was reduced to 33.3% if symptoms lasted more than six months before initiating treatment.	
Shirah HA et al., 2022 [19].	Non-RCT	539	Saudi Arabia	19.3-30.7	397 (73.7%) patients achieved recovery after conservative treatment. The duration of treatment until full recovery was 3.5 weeks.	Sitz baths showed promising results when included in the conservative management of anal fissures. The study recommends further studies to look into the role of sitz baths in the conservative management of anal fissures.	
Kaur H et al., 2022 [20].	Non-RCT	60	India	28-41	60 patients were divided into two groups of 30 patients in each group. They were monitored for pain relief using a scoring system and recurrence rates. In the conservative group, 13 patients reported low pain, three patients reported moderate pain, and 14 patients reported extreme pain. The median time for healing was four weeks. Twenty patients had recurrences after 6 months. In the surgical group, 26 patients had low pain, two patients had moderate pain, and two patients had extreme pain. The median time for healing was three weeks. Two patients had recurrences after six months.	Bilateral LSIAS surgery is more effective than the conservative treatment of anal fissures in relation to pain relief and recurrence. The study concludes that bilateral LSIAS surgery results in higher chances of early recovery and pain relief, a better quality of life, and fewer risks of progression to chronic anal fissures. However, it does not rule out the role of the conservative treatment, which showed good outcomes in 50% of the patients. Yet, the recurrence rates were much higher (66%).	
Bhasker V et al., 2019 [21].	Non-RCT	60	India	20-50	Patients were divided into two groups of 30 each, and healing rates were monitored. Medical: 24 recovered in three weeks, and 25 recovered in four, six, and eight weeks. Surgical: 23 patients recovered in three weeks, 26 patients recovered in four weeks, and 30 patients recovered in six and eight weeks.	Diltiazem gel with sitz bath and laxative showed improving results in the management of anal fissure. Lateral internal sphincterotomy is still the gold standard treatment, especially in cases resistant to conservative treatment.	

Setting

Five randomized control trials were conducted: one was in Denmark in 1983; one in India in 2022; one in Iran in 2022; one in an unspecified country in 2010, and another one in an unspecified country in 2015. Five non-randomized control trials were conducted: one in India in 2019 and two in 2022; one in Egypt in 2017; and one in Saudi Arabia in 2022. One systematic review was conducted in Singapore in 2010.

Outcomes Measured

This review measures specific outcomes from the selected studies. This includes pain relief, healing time of the fissure, comparing the studied treatment to the gold standard lateral internal sphincterotomy, and side effects of the treatment. All the outcomes are related to sitz baths.

Discussion

This section explores the results and effectiveness of using sitz baths as a treatment of choice. This study will assess the effectiveness of sitz baths in relieving pain and healing wounds quickly, comparing them to the gold standard of lateral internal sphincterotomy as a surgical treatment and the complications associated with the treatment options.

Analgesia

The role of sitz bath as analgesia was explored in four randomized control trials, two non-randomized control trials, and one systematic review. The studies showed conflicting evidence for the use of sitz baths for the management of pain in patients with anal fissures.

Gogna S’ 2015 study compared the conservative treatment modalities between using a sitz bath and psyllium husk, glyceryl trinitrate, and lidocaine. The study demographics included 16-60-year-old patients, with the male-to-female ratio being 1.6:1, presenting to the clinic with acute anal fissures. During the first week of follow-up, 88% of the group with the sitz bath showed symptomatic relief. As the study mentions, warm sitz baths are effective in treating sphincter spasms [11].

Jensen SL’s study in 1983 shows a similar study was conducted using 96 patients that were presented to the clinic with acute anal fissures. The study compared using the conservative treatments of lignocaine, hydrocortisone, sitz bath, and bran. During the first two weeks, the group treated with sitz bath with bran showed a lower pain score as compared to the other groups. But after the third week, they all had the same pain score [12].

In Maestre Y’s study, the analgesic properties of hot and cold sitz baths were explored. The study was limited in the number of patients as well as the outcome assessment. The study concluded that there was no significant difference between both groups. However, both groups showed an overall reduction in pain over seven days [13].

Rathore RK’s study in 2019, compares the effects of using warm sitz baths against using warm sitz showers. The study was conducted on fifty anal fissure patients, allocated into two treatment groups. The study found that within seven days, both groups showed improvement in symptoms and a reduction in pain, with patients using a warm sitz shower showing a more significant improvement. The study also found that patients preferred a warm sitz shower over a warm sitz bath. This study introduces a new form of warm bath that is more efficacious and satisfactory to patients. However, more studies need to be conducted regarding this new method of treatment [14].

Lang DS's systematic review (2010), conducted in Singapore, evaluates four randomized control trials on the effectiveness of managing patients with anorectal disorders. This study is nonspecific to anal fissures but does include them. The type of intervention used was mainly sitz bath, with the possibility of other conservative treatment tools. The study utilized a pain score criteria, which showed that patients using a sitz bath experienced less pain as compared to patients not being treated. However, both groups showed improvement in pain symptoms, and the difference was not of any statistical significance. This review does not support the use of a sitz bath for the management of anal fissure pain [15].

Motie MR’s study in 2015 looked at 190 patients that were referred to the surgical clinic for chronic fissures. The patients were randomly divided into three groups. Each group was treated with either nitroglycerin with a sitz bath, diltiazem with a sitz bath, or lateral internal sphincterotomy. The patients were followed up for eight weeks, and their response in terms of analgesic properties was recorded. Patients treated with nitroglycerin and sitz bath achieved a rating of 77% pain relief. Patients treated with diltiazem with a sitz bath achieved a rating of 83% pain relief. Both groups of patients reached their respective ratings in eight weeks. However, the group treated with lateral internal sphincterotomy achieved a 100% score in pain relief within only four weeks. Although the study highlights the limitations of conservative treatments, it is worth noting that pain relief was achieved in up to 83% of patients receiving conservative treatment with a sitz bath [16].

Randomized control trials are considered strong research sources, second only to systematic reviews and meta-analyses. All randomized control trials concluded that using a sitz bath is beneficial in terms of analgesia in patients with anal fissures. Both non-randomized control trials were consistent with the randomized control trial findings, supporting the use of sitz baths for analgesic effects. However, the systematic review did not support the use of a sitz bath for its analgesic properties, as it concluded there was no statistical significance in the trial and control groups. Given all the evidence presented in this study, the use of a sitz bath for its analgesic properties is supported in patients with anal fissures.

Healing Time

The properties of healing weigh in on the option of using sitz baths as a treatment modality for anal fissures. This property has been explored by four randomized control trials, two non-randomized control trials, and one systematic review.

Kaushal M’s study in 2022 looked at 90 patients. The patients were divided into three groups: patients treated with lidocaine pomade and sitz bath; zinc oxide and sitz bath; and lateral internal sphincterotomy. The outcome was measured based on their recovery rate. The study showed that lateral internal sphincterotomy was the superior treatment, with a 100% recovery rate. In contrast, zinc oxide pomade with a sitz bath had a recovery rate of 23.33%, and lidocaine pomade with a hot sitz bath had a recovery rate of 26.66%. The study claims no statistical difference in non-operative management compared to surgical treatment [17].

Gonga S' study had 75 patients divided into three groups: one group received psyllium husk and sitz baths; the other received lignocaine; the third group received glyceryl trinitrate. In the second week, healing was achieved in 72% of the patients that received psyllium husk and sitz baths, 36% of the patients that received lignocaine, and 76% of the patients that received glyceryl trinitrate. At the end of the three-week follow-up, healed fissures were found in 80% of the group that received psyllium husk and sitz baths, 40% of the groups that received lignocaine, and 84% of the group that received glyceryl trinitrate. The study shows that a sitz bath is as efficacious in healing anal fissures as glyceryl trinitrate. However, 20% of the patients treated with sitz baths had unhealed anal fissures and would probably require surgical intervention [11].

Jensen SL's study showed that healing in the first two weeks of treatment was significantly greater in the group that received a sitz bath with bran, compared to the groups using hydrocortisone and lidocaine ointments. After three weeks of treatment, healing rates were similar across all groups. This study shows the efficiency of using conservative management to heal anal fissures. Moreover, the study highlights the healing time in patients compliant with sitz baths as opposed to other treatments [12].

Motie MR’s study followed up with patients, assessing the healing of the fissures for up to eight weeks. The patients receiving conservative treatment with sitz baths showed 74%-83% healing in anal fissures. These numbers are consistent with Gonga S' study [11]. It also shows the limitations of conservative treatment, with about 20% of patients requiring further management, possibly even surgical intervention [16].

In Emile SH’s study in 2017, the effects of conservative treatments were looked at by comparing acute and chronic fissures. The study included 65 patients with anal fissures. The conservative management consisted of laxatives, sitz baths, and glyceryl trinitrate. The findings were similar to Gonga S’ and Motie MR’s studies, as they show that 80% of acute anal fissures healed with conservative treatment. However, only 33% of patients achieved complete healing for chronic fissures lasting more than six months. This study demonstrates that sitz baths have limited use in chronic fissure treatment [18].

Shirah HA’s study in 2022 looked at the effects of warm sitz baths with regard to anal fissures. The study included 539 patients followed up in the outpatient department. The patients were given instructions on how to use sitz baths and then assessed in the follow-up. The results were consistent with other studies mentioned in this review. 73.7% of patients had achieved healing after complying with the conservative treatment. The average time to achieve healing was 3.5 weeks. The study concludes that sitz baths should be considered as the main treatment modality for anal fissures [19].

As for the systematic review by Lang DS, the patient’s recovery was assessed at the end of the four-week trial. Both groups with sitz baths and those without sitz baths showed a healing rate of more than 90%. The difference between the two groups was 1.4% and was not considered significant enough to justify using a sitz bath to heal anal fissures. The review suggests using water sprays to improve hygiene [15].

Overall, wound healing is the most vital assessment for patients receiving treatment for anal fissures. The findings of the studies collectively display conflicting evidence concerning the efficacy of sitz baths in wound healing. However, much of the evidence supports the sitz bath as a primary tool to heal anal fissures. Most studies reported a healing rate of 80% within six weeks of starting treatment.

Compared to Lateral Internal Sphincterotomy

Kaushal M’s study compares the inclusion of a sitz bath to conservative therapy, with lateral internal sphincterotomy as the gold standard treatment for anal fissures. As mentioned above, the recovery rates vary between the conservative and surgical groups, with the conservative treatment having a 23.33% to 26.66% recovery rate, while the surgical treatment has a 100%. When compared to conservative treatments, the comparison supports lateral internal sphincterotomy as a treatment modality [17].

Motie MR’s study, similar to Kaushal M’s study, compares conservative therapy with the gold standard of lateral internal sphincterotomy. However, the results differ as the conservative treatment shows a recovery rate of 74% to 83% in conservative treatments as compared to 94% in the lateral internal sphincterotomy. The comparative difference in Motie MR's results is still significant in favor of lateral internal sphincterotomy, but not as dramatic as Kaushal M’s results [15].

Kaur H’s study in 2022 involved 60 patients divided randomly into two groups. One group received conservative treatment involving diltiazem with sitz baths, and the other group had bilateral lateral subcutaneous internal anal sphincterotomy. The results demonstrate that LSIAS is superior to conservative treatment. It also mentions that the recovery rates are higher with conservative treatments, at around 66%. This study does not exclude the role of conservative therapy in anal fissures [20].

Bhasker V’s study in 2019 involved 60 patients from a surgical clinic who had been suffering for more than two months from anal fissures. Two groups were formed, with 30 patients in each group. One group received conservative treatment of oral laxatives, diltiazem, and sitz baths. The other group received the surgical treatment of lateral internal sphincterotomy, followed by sitz baths and laxatives. The Sitz bath is included in both groups, making it hard to distinguish the effect of this treatment. In the first three weeks, the conservative group showed improvement in symptoms and healing compared to the surgical group, but it was not statistically significant. However, at the end of the trial, five patients did not show improvement, leaving an 83.3% success rate. In the surgical group, all patients were healed. This study shows that the effects of conservative management, even with sitz baths, are limited in their success rate [21].

All studies in this article hold the gold standard of surgical lateral internal sphincterotomy as the superior treatment of anal fissures. Most studies do not rule out the role of conservative therapy in treating anal fissures. Conservative treatment with sitz baths has been proven to successfully treat most cases, up to 80% in some studies. When considering the risks of surgery and anesthesia, as well as the costs associated with the surgery, conservative management offers significant advantages in treatment options.

Side Effects

Complications with medications have been reported in these articles.

Jensen SL’s study uses hydrocortisone to treat anal fissures. One such adverse reaction was recorded when one homosexual patient treated with hydrocortisone ointment later developed extensive herpetic lesions locally. In the group where a sitz bath with bran was used, 40.6% of the patients had abdominal distention and exhibited flatulence to various degrees. Yet, none of the patients stopped the treatment. The group treated with lignocaine did not experience any side effects [12].

Motie MR’s study showed that 17 patients using nitroglycerin experienced headaches. Three of the patients claimed it to be severe. No side effects were seen in the group using diltiazem. Two patients in the group receiving lateral internal sphincterotomy reported gas incontinence after surgery. The issue was resolved during the fourth-week visit. After one year of therapy, 17 patients had a recurrence, as compared to the surgical group, where they had no recurrences [16].

Emile SH’s study showed that 30% of patients developed headaches during treatment, while 25% developed postural hypotension and palpitation. Headaches were significantly higher with acute as compared to chronic anal fissures. No significant difference between acute and chronic fissures was noted regarding postural hypotension [18].

None of the studies showed any side effects relating to sitz baths. However, if used in conjunction with glyceryl trinitrate, there is a high risk of developing headaches and postural hypotension. If used as an adjuvant with bran, patients could experience flatulence and distension. With lateral internal sphincterotomy, most studies show that patients had temporary gas incontinence which generally resolved four weeks after surgery.

Limitations

The review was limited in terms of the articles selected, as this study only included free-text research. This review could have been more specific regarding the type of fissure, whether it was chronic or acute, and the type of sitz bath, whether it was cold or hot, or as spray or bath. The main limitation of this study was the limited amount of research regarding the specific use of a sitz bath. Given that sitz baths are not conventionally used alone to treat the patient, most studies look at conservative methods, including medical treatment with sitz baths, which limits the study's accuracy. Studies should include different conservative methods like diltiazem or lidocaine.

## Conclusions

This systematic review focused on the feasibility of using sitz baths as an effective treatment of anal fissures. Based on the overall results of the articles, this study supports the use of sitz baths to treat anal fissures. In line with the results presented, the study finds sitz baths to have analgesic properties as well as a good recovery rate. However, when compared to lateral internal sphincterotomy, there is a significant difference in the recovery rate at the end of the treatment, with lateral internal sphincterotomy being superior. This is consistent with the existing literature. As for the side effects, none have been reported from using the sitz bath. The side effects experienced in the conservative treatment mostly resulted from using adjuvants in sitz baths. This paper aims to encourage a more conservative approach, using fewer resources, less invasive, and with minimal complications to a common clinical condition. Questions that arose during the study revolved around the use of bidet hygiene as an alternative treatment. Further studies are recommended to strengthen the knowledge base around this topic.
